# Hypotension Prediction Index with non-invasive continuous arterial pressure waveforms (ClearSight): clinical performance in Gynaecologic Oncologic Surgery

**DOI:** 10.1007/s10877-021-00763-4

**Published:** 2021-10-07

**Authors:** Luciano Frassanito, Pietro Paolo Giuri, Francesco Vassalli, Alessandra Piersanti, Alessia Longo, Bruno Antonio Zanfini, Stefano Catarci, Anna Fagotti, Giovanni Scambia, Gaetano Draisci

**Affiliations:** 1 Department of Scienze dell’Emergenza, Anestesiologiche e della Rianimazione, IRCCS Fondazione Policlinico A. Gemelli, Rome, Italy; 2 Edwards Lifesciences Italia, Irvine, CA USA; 3 Department of Scienze della Salute della Donna, del Bambino e di Sanità Pubblica, IRCCS Fondazione Policlinico A. Gemelli, Rome, Italy

**Keywords:** Intraoperative hypotension, Hemodynamic monitoring, Volume clamp method, Machine learning, Hypotension prediction, Gynaecologic Oncologic Surgery

## Abstract

Intraoperative hypotension (IOH) is common during major surgery and is associated with a poor postoperative outcome. Hypotension Prediction Index (HPI) is an algorithm derived from machine learning that uses the arterial waveform to predict IOH. The aim of this study was to assess the diagnostic ability of HPI working with non-invasive ClearSight system in predicting impending hypotension in patients undergoing major gynaecologic oncologic surgery (GOS). In this retrospective analysis hemodynamic data were downloaded from an Edwards Lifesciences HemoSphere platform and analysed. Receiver operating characteristic curves were constructed to evaluate the performance of HPI working on the ClearSight pressure waveform in predicting hypotensive events, defined as mean arterial pressure < 65 mmHg for > 1 min. Sensitivity, specificity, positive predictive value and negative predictive value were computed at a cutpoint (the value which minimizes the difference between sensitivity and specificity). Thirty-one patients undergoing GOS were included in the analysis, 28 of which had complete data set. The HPI predicted hypotensive events with a sensitivity of 0.85 [95% confidence interval (CI) 0.73–0.94] and specificity of 0.85 (95% CI 0.74–0.95) 15 min before the event [area under the curve (AUC) 0.95 (95% CI 0.89–0.99)]; with a sensitivity of 0.82 (95% CI 0.71–0.92) and specificity of 0.83 (95% CI 0.71–0.93) 10 min before the event [AUC 0.9 (95% CI 0.83–0.97)]; and with a sensitivity of 0.86 (95% CI 0.78–0.93) and specificity 0.86 (95% CI 0.77–0.94) 5 min before the event [AUC 0.93 (95% CI 0.89–0.97)]. HPI provides accurate and continuous prediction of impending IOH before its occurrence in patients undergoing GOS in general anesthesia.

**Supplementary Information:**

The online version contains supplementary material available at 10.1007/s10877-021-00763-4.

## Introduction

Intraoperative hypotension (IOH) represents a common event during general anaesthesia, with a reported incidence, varying with the chosen threshold, from 5 to 99% [1]. Intraoperative mean arterial blood pressure (MAP) values below 65 mmHg are associated with myocardial injury, acute kidney injury and death, proportionate to hypotension severity and duration [2–6].

Gynaecological oncological surgery (GOS) for cancer mass reduction is carried out through laparoscopy or laparotomy and is often associated with unstable hemodynamics and significant blood loss [7, 8]. Hypotension during GOS is common, and as it is associated with the potential for harm, it requires prompt evaluation and treatment [8]. Extensive fluid resuscitation in peritoneal cancer patients is associated with a poor postoperative outcome and avoiding fluid overload is recommended [7]. On the other hand, avoiding low blood pressure preserves organ perfusion [6].

Machine learning, a discipline within computer science used to analyse large data sets and to develop predictive models, has evident applications in health care [9–13]. Several attempts to use algorithms as an aid in anaesthesiology practice recently received renewed attention, with the aim of optimizing patients’ perioperative status, primarily focusing on detection of early hemodynamic instability and prediction of hypotension [14, 15].

The Hypotension Prediction Index—HPI (Edwards Lifesciences, Irvine, USA) is an algorithm based on the complex analysis of features in high fidelity arterial pressure recordings developed to detect the interconnection signatures in the arterial pressure waveform when hypotension is impending [16]. The algorithm uses a large analysis of interaction effects to assess compensatory mechanisms and capture the cross-correlational changes among thousands of automatically derived hemodynamic features that herald the onset of hypotension [16]. HPI is a unitless number that ranges from 0 to 100, and as the number increases, the likelihood or risk of a hypotensive event (defined as a MAP < 65 mmHg for more than 1 min) occurring in the near future increases [16]. A validation study conducted on surgical patients reported high sensitivity and specificity of HPI for predicting hypotension 5, 10 and 15 min before the event occurred [17]. The development of the algorithm was based on invasive arterial line waveform data; however, only a small fraction of patients having noncardiac surgery requires invasive arterial monitoring [18, 19].

The aim of this study was to assess the diagnostic ability of the HPI algorithm derived from non-invasive ClearSight system in predicting impending hypotension in patients scheduled for elective major GOS.

## Materials and methods

This is a retrospective analysis of data collected during a limited period of time in which new hemodynamic monitoring sensors (ClearSight) were evaluated. The study was approved by the Internal Ethic Committee (ID 3664, protocol number 10077/21). Written informed consent to treatment of data was obtained from the patients. This manuscript adheres to the applicable STROBE guidelines.

Data were gathered from patients who had perioperative monitoring with the HemoSphere platform with ClearSight non-invasive hemodynamic monitoring and with the HPI software enabled at IRCCS Policlinico Agostino Gemelli Foundation of Rome, Italy. Data were collected from 1 December 2019 to 31 January 2020.

All patients scheduled for GOS who had intact complete data sets available for analysis were enrolled in the study. Exclusion criteria were: < 18 years of age, an American Society of Anesthesiologists score (ASA) > 3, significant cardiac arrhythmias or aortic regurgitation, permanent atrial fibrillation, coagulation disorders, emergency surgery, preoperative infection and patient’s refusal to treatment of personal data.

Standard monitoring (Life Scope TR, Nihon Kohden Co, Tokyo, Japan) included a 5-lead electrocardiogram, pulse oximetry, non-invasive blood pressure (NIBP) and eventual invasive blood pressure (IBP). In addition to standard monitoring, all patients had a non-invasive hemodynamic monitoring with ClearSight (Edwards Lifesciences, Irvine, CA).

**Fig. 1:**
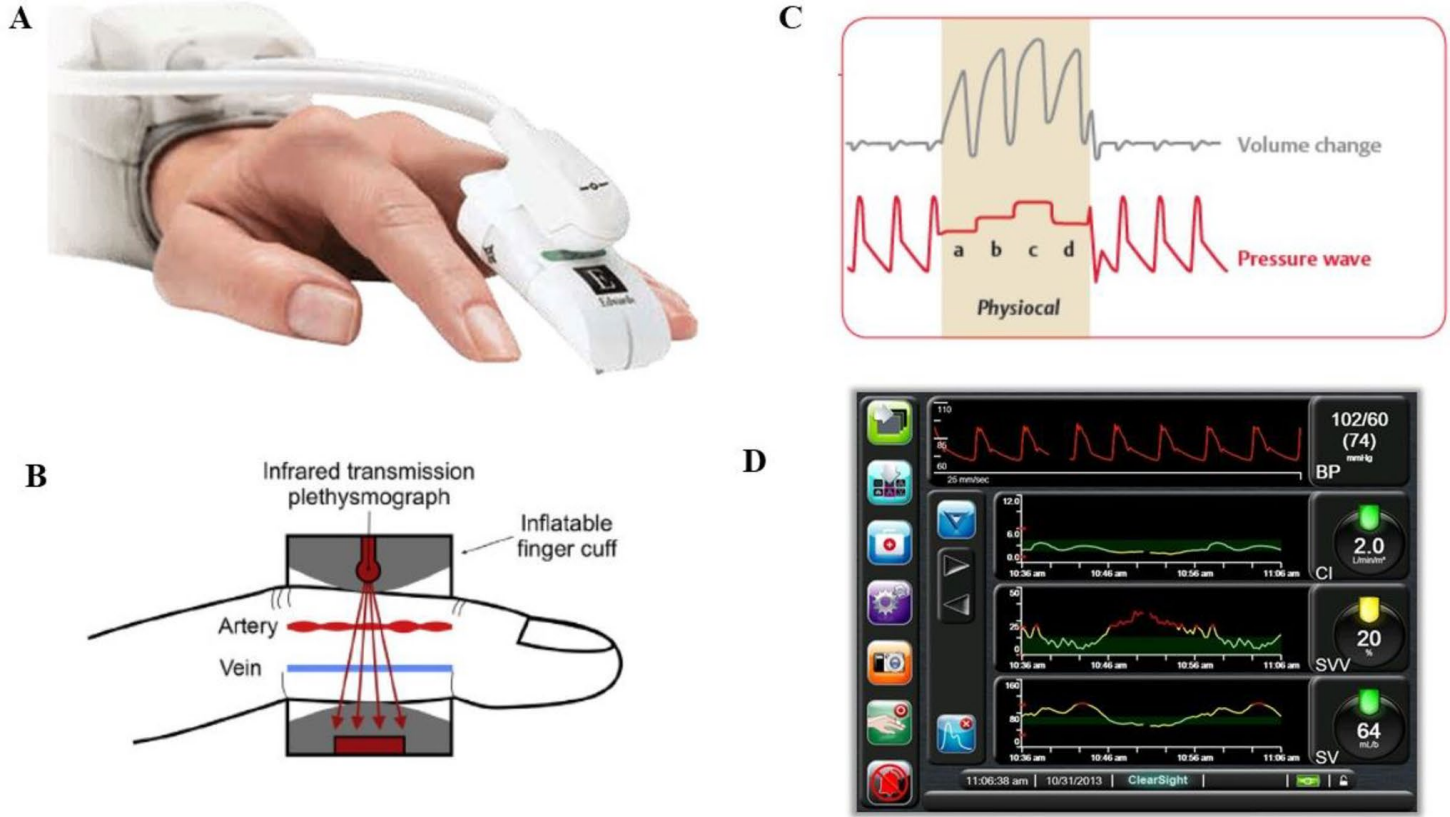
**A** The non-invasive ClearSight finger cuff. **B** Volume clamp—vascular unloading technique. The inflatable finger cuff measures the diameter of the finger artery with an integrated infrared transmission plethysmograph. This leads to high-frequent adjusts of the cuff pressure to keep the blood volume in the finger artery constant throughout the cardiac cycle (**C**). From the pressure adjustments needed to maintain a constant blood volume in the finger artery the arterial blood pressure waveform can be derived and analysed to estimate arterial blood pressure and cardiac output. **D** Example of HemoSphere hemodynamic monitor screen

After arriving at the operating theatre, NIBP measurement using an automated digital sphygmomanometer on the right arm was started and the ClearSight system was attached to a finger of the left arm of the patients (Fig. 1, panel A). We connected the ClearSight monitor with an interface cable to the patient monitor. In patients requiring IBP monitoring an arterial cannula was placed contralateral to the ClearSight cuff. The ClearSight reference system was zeroed at the level of the right atrium.

The attending anesthesiologist was blinded to the advanced hemodynamic parameters from the ClearSight system (including also HPI predictions and alarms), except for the continuous arterial pressure value read from the finger cuff (CS-BP), which was reported on the main monitor. NIBP measurement using the automated digital sphygmomanometer was performed at 5-min intervals.

A large bore i.v. catheter was inserted in a forearm vein, and Cefazoline 2 gr, Dexamethasone 4 mg and Omeprazole 40 mg were administered. General anesthesia was induced with sufentanil 0.2 mcg/kg (ideal body weight), propofol 2 mg/kg (actual body weight), and rocuronium 0.6 mg/kg (ideal body weight). During surgery, patients were positioned in Trendelenburg position with both arms spread out on arm-positioning devices. Anesthesia was maintained with Sevoflurane to maintain a Bispectral Index value between 40 and 50. Additional boluses of sufentanil and rocuronium were administered when needed.

For therapy purposes we defined hypotension as an absolute value of CS-BP MAP < 65 mmHg. Incidence and duration of hypotensive episodes and interventions were registered. Bradycardia was defined as a heart rate (HR) < 60 bpm. Clinicians were free to manage haemodynamic state of the patients using any type of intravenous fluids, vasopressors, and inotropes, according with their personal judgement and institutional clinical practice.

All data were downloaded from the HemoSphere monitor, including HPI, CS-MAP, CS systolic arterial pressure, CS diastolic arterial pressure. All downloaded data consisted of 20-s interval averaged data points. Data were transferred to a computer for analysis via an USB drive. Every file was appointed with an automated generated code by the machine and was identifiable by an ID number contained within it.

The HPI algorithm estimates the probability of occurrence in the near future of a hypotensive event taking the arterial pressure waveform as the input to compute an index value that ranges between 0 and 100. In this study, instead of invasive arterial waveform data, we used the non-invasive arterial pressure waveform of ClearSight. The alarm threshold for HPI is fixed at 85, and it was silenced, as all the other ClearSight variables.

In the HemoSphere monitor, poor quality arterial waveforms were automatically detected by the arterial waveform processing algorithms and excluded from the computation of the 20-s averages.

### Statistical analysis

A data analysis and statistical plan was designed after the data were accessed. An a priori sample size calculation was not performed.

Normally distributed continuous data are presented as mean (SD), non-normally distributed data as median (25th–75th percentile). Categorical data are presented as n (%). Normality of data distribution was assessed with the Shapiro–Wilk test and visually with histograms; the equality of variance was verified with the variance ratio test. P values < 0.05 were considered statistically significant.

Hypotensive events (defined as a MAP < 65 mmHg for > 1 min) were analysed in terms of number, absolute duration, area under the threshold of 65 mmHg, and time-weighted average under the threshold, calculated as area under the threshold/duration of monitoring, similar to as described by Maheshwari et al. [20]. The number of HPI alarms was assessed. A non-hypotensive event was calculated by identifying a 30-min continuous section of data points such that the section was at least 20 min apart from any hypotensive event, and all data points in that section showed MAP > 65 mmHg [16, 17]. A non-event, or negative data point, was the centre point of the non-hypotensive event [16, 17].

Receiver operating characteristic (ROC) curve analysis was used to evaluate the performance of the HPI algorithm working on the ClearSight blood pressure waveform. Sensitivity was reported on the y-axis and 1-specificity on the x-axis. The area under the curve (AUC) estimating the ability of HPI to discriminate between hypotensive versus non hypotensive events at 5, 10 and 15 min before the actual event occurred was computed. Sensitivity, specificity, positive predictive value, and negative predictive value were computed at a cutpoint, identified as the value that minimizes the difference between sensitivity and specificity [16].

A true positive was considered any hypotensive event (MAP < 65 mmHg for at least 1 min) with HPI value greater than or equal to a chosen threshold (the cutpoint) at 5, 10, or 15 min. A true negative was any non-event data point (MAP > 65 mmHg) with HPI value less than the chosen threshold. Sensitivity is the ratio of true positives to all events. Specificity is the ratio of true negatives to all non-events. Positive predictive value was calculated as the number of true positive events divided by all positive ones. Negative predictive value was the number of true negative events divided by all negative ones.

ROC analysis was also used to evaluate the performance of the change of CS-MAP (ΔMAP) to predict hypotension 5, 10 and 15 min before its occurrence.

In the ROC analysis, the repeated measures from the same patient were compensated using the bootstrapping method: 28 patients were randomly chosen from the total 28 patients with replacement. This process was repeated 2000 times from which the standard error was calculated. The bootstrap CI was calculated as a 95% asymptotic confidence interval.

Data analysis was performed using STATA MP 15.1 (Stata Corp) for Windows, Microsoft Excel, Matlab (The MathWorks Inc., Natick, MA, USA), and Acumen Analytics software (Edwards Lifesciences, Irvine, CA).

## Results

**Fig. 2:**
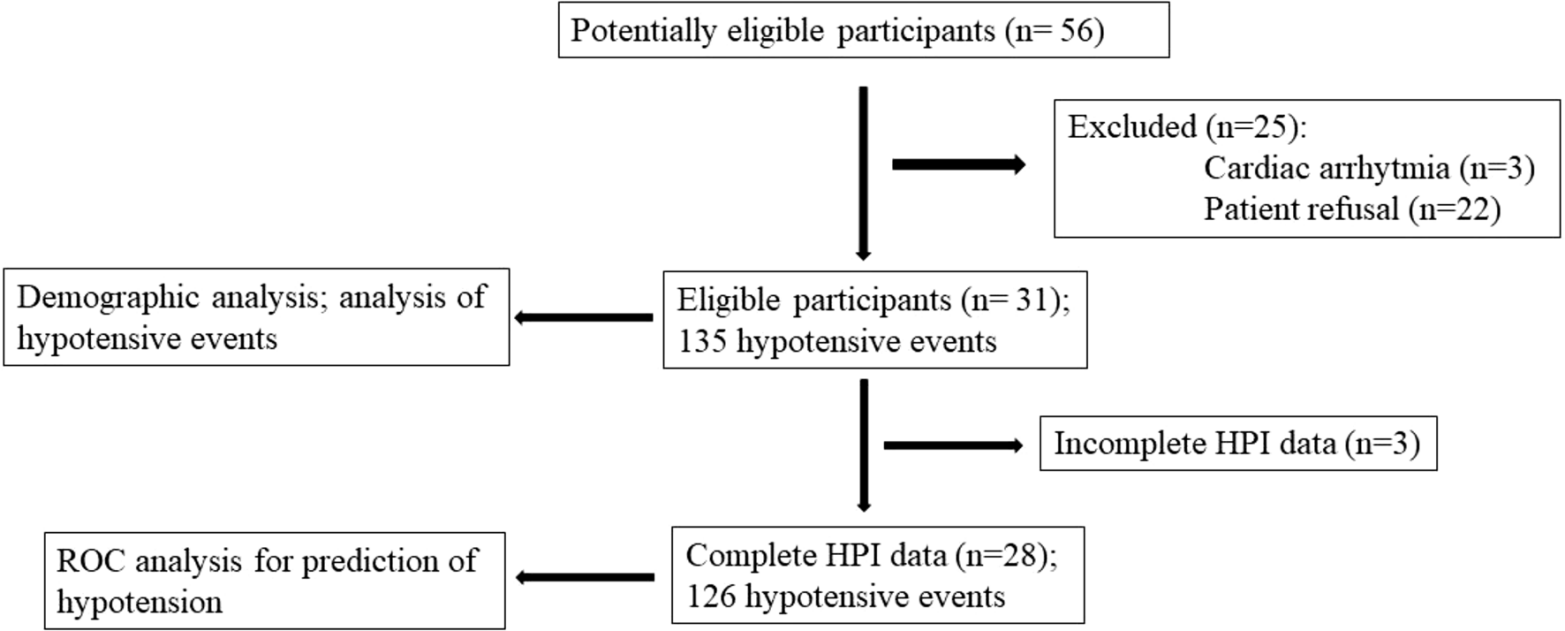
Flow of participants through the study. The study time frame is from the beginning of hemodynamic monitoring until the end of surgery. *HPI* Hypotension Prediction Index, *ROC* receiver operating characteristic

Fifty-six patients undergoing major GOS from 1 December 2019 to 31 January 2020 were screened during study recruitment. Twenty-five cases were not included because they did not meet the inclusion criteria. Thirty-one patients with a mean age of 50 (± 10.44) years were included in the analysis. In 3 cases data were incomplete for technical problems with HPI monitoring and were excluded from ROC analysis. The data collected from remaining 28 women were complete (Fig. 2).

Demographic parameters and kind of surgery are shown in Table 1. The median monitoring time per patient was 194 (150–344) minutes. Twenty-four of 31 subjects (77.4%) had at least 1 hypotensive event. One hundred thirty-five hypotensive events were detected and the median number of events per patient was 4 (1–6.75), with a median duration of 2.67 (1.33–4.67) minutes per event. The median time spent in hypotension per procedure was 10 (1.5–35.5) minutes, with a median area under the threshold of 65 mmHg of 47.5 (2.8–177.7) mmHg per minutes, and a median time-weighted average area under the threshold of 0.18 (0.01–0.71). Nine patients experienced a hypotensive event with MAP under 50 mmHg, with lowest MAP of 39 mmHg.

**Table 1:** *SD* standard deviation, *IQR* interquartile range, *ASA* American Society of Anesthesiologists (1: a healthy person; 2: a patient with mild systemic disease; 3: a patient with severe systemic disease; and 4: a patient with severe systemic disease that is a constant threat to life), *TWA* time-weighted average, *MAP* mean arterial pressure

Age—years (mean ± SD)	50 (± 10.44)
Height—cm (mean ± SD)	163(± 6)
Weight—kg (mean ± SD)	67 (± 13)
ASA classification—n (%)	
1	5 (16%)
2	19 (61%)
3	7 (23%)
4	0
Type of GOS—n (%)	
Ovarian cancer	9 (29%)
Endometrial cancer	12 (39%)
Cervical cancer	10 (32%)
Surgical approach—n (%)	
Laparoscopic	20 (65%)
Laparotomy	9 (29%)
Conversion	2 (6%)
Monitoring time per patient—minutes (median [IQR])	194 [150, 344]
Number of patients with hypotension—number (%)	24 (77.4%)
Number of hypotensive events per patient—number (median [IQR])	4 [1, 8]
Duration of cumulative hypotension—minutes (median [IQR])	10 [1.5, 35.5]
TWA (MAP < 65 mmHg) per patient—mmHg (median [IQR])	0.18 [0.01, 0.71]

**Fig. 3:**
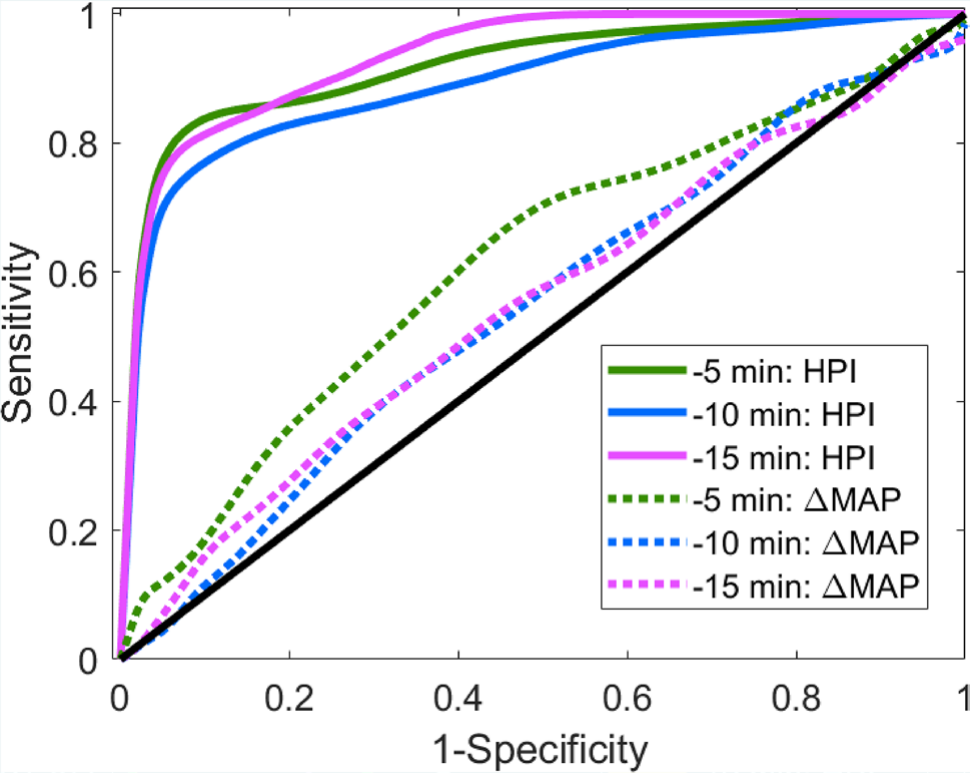
Receiver operating characteristic curves for HPI (Hypotension Prediction Index) and ΔMAP (changes in mean arterial pressure) over the preceding 15 min for predicting hypotension 5, 10 and 15 min before its occurrence. ROC is a plot of true positive rate (sensitivity) and false positive rate (1—specificity) at HPI values from 0 to 100

ROC curves displaying the ability of HPI to predict hypotension 5, 10 and 15 min before its occurrence are shown in Fig. 3. The AUC for the prediction of hypotension 15 min before the event was 0.95 (95% CI 0.89–0.99); at the cutpoint of 44.3, sensitivity was 0.85 (95% CI 0.73–0.94) and specificity 0.85 (95% CI 0.74–0.95) with a positive predictive value of 0.75 (95% CI 0.43–0.92) and a negative predictive value of 0.91 (95% CI 0.81–0.98). The positive data points analysed were 28, the negative data points 49.

The AUC for prediction of hypotension 10 min before the event was 0.9 (95% CI 0.83–0.97); at the cutpoint of 41.6, sensitivity was 0.82 (95% CI 0.71–0.92), specificity was 0.83 (95% CI 0.71–0.93) with a positive predictive value of 0.79 (95% CI 0.56–0.93) and a negative predictive value of 0.85 (95% CI 0.73–0.94). The positive data points analysed were 41, the negative data points 49.

The AUC for prediction of hypotension 5 min before the event was 0.93 (95% CI 0.89–0.97); at the cutpoint of 44.1, sensitivity was 0.86 (95% CI 0.78–0.93), specificity was 0.86 (95% CI 0.77–0.94) with a positive predictive value of 0.88 (95% CI 0.77–0.96) and a negative predictive value of 0.82 (95% CI 0.70–0.91). The positive data points analysed were 64, the negative data points 49.

Results of ROC analysis are showed in Table 2. The results of ROC analysis evaluating the performance of the ΔMAP over the last 15 min to predict hypotension at 5, 10, and 15 min from the occurrence of the hypotensive event are shown in Fig. 2. AUC, sensitivity, specificity, and cutpoints are shown in Table 2.

**Table 2:** Receiver operating characteristic analysis for Hypotension Prediction Index (HPI) and changes in mean arterial pressure (ΔMAP) to predict hypotension 5, 10 and 15 min before its occurrence

Time	AUC [95% CI]	Sensitivity [95% CI]	Specificity [95% CI]	Positive predictive value [95% CI]	Negative predictive value [95% CI]	Optimal value
HPI						
5 min	0.93 [0.89, 0.97]	0.86 [0.78, 0.93]	0.86 [0.77, 0.94]	0.88 [0.77, 0.96]	0.82 [0.70, 0.91]	44.1
10 min	0.90 [0.83, 0.97]	0.82 [0.71, 0.92]	0.83 [0.71, 0.93]	0.79 [0.56, 0.93]	0.85 [0.73, 0.94]	41.6
15 min	0.95 [0.89, 0.99]	0.85 [0.73, 0.94]	0.85 [0.74, 0.95]	0.75 [0.43, 0.92]	0.91 [0.81, 0.98]	44.3
Δ MAP						
5 min	0.62 [0.51, 0.73]	0.59 [0.43, 0.73]	0.61 [0.49, 0.72]	0.38 [0.25, 0.53]	0.78 [0.66, 0.88]	1.85
10 min	0.55 [0.50, 0.62]	0.54 [0.44, 0.64]	0.53 [0.45, 0.63]	0.25 [0.14, 0.36]	0.8 [0.68, 0.91]	1.16
15 min	0.55 [0.50, 0.66]	0.55 [0.43, 0.68]	0.54 [0.42, 0.69]	0.21 [0.09, 0.33]	0.84 [0.74, 0.95]	0.74

*AUC* area under the curve, *CI* confidence interval. Sensitivity and Specificity results are taken at the optimal value for HPI in ROC (cutpoint value)

## Discussion

This study demonstrates that the HPI algorithm working with non-invasive arterial pressure waveforms monitoring (ClearSight) can predict hypotension with high accuracy in patients undergoing major GOS up to 15 min before the event.

Blood pressure is one of the main determinants of organ perfusion. Profound IOH is common in patients undergoing surgery and is associated with hypoperfusion and organ failure. To date, treatment of hypotension is “reactive”, and the physician acts only after low blood pressure values have already occurred.

Recent studies have suggested that the prodromal stage of hemodynamic instability is characterized by complex changes in different physiologic variables, reflecting altered compensatory mechanisms, and resulting in unique dynamic signatures in arterial waveforms heralding the occurrence of the vast majority of hypotensive events [14–17]. Prediction of impending hypotension could allow the clinician to act in a “pro-active” manner even before the blood pressure drops, theoretically reducing the incidence and duration of hypotensive episodes. Recently there has been growing interest in the application of artificial intelligence and Machine Learning in medicine [14, 15]. Many machine learning algorithms have increasingly been used to analyse biosignal waveforms from patient monitoring systems and to predict medical conditions, such as major complications after surgery, heart failure, acute respiratory distress syndrome, sepsis, COVID-19, ameliorating decisional process and personalization of treatment [7–13].

### Hypotensive load in GOS

Approximately two thirds of our patients underwent laparoscopic surgery with Trendelemburg position up to 30°, which is well known to increase MAP, while one third of the patient underwent a laparotomic intervention with minimal to none Trendelemburg position. The hypotensive load of our patients measured as median TWA-MAP < 65 mmHg (0.18 [0.01, 0.71] mmHg) is similar to that of other cohorts (0.14 [0.03, 0.39] mmHg and 0.05 [0.00, 0.22] mmHg reported in the papers of Maheshwari, and 0.3 [0.0,1.2] mmHg in the work of Wijnberge) [18–20]. Expected odds are justified by different types of surgery, patient’s characteristics, and institutional protocol for hemodynamic management.

### HPI applied to invasive and non-invasive arterial waveform: any difference?

There is growing evidence regarding the performance of the HPI applied to invasive arterial waveforms in large cohort of patients [21, 22]. More recently, this technology has been applied to non-invasive arterial pressure waveforms derived from ClearSight [18, 19]. In Supplementary Table 1 we compare the available evidence of HPI performance working either with invasive or non-invasive arterial pressure waveform. As shown, the HPI algorithm has an excellent overall performance, regardless of cohorts and data source, with AUC ≥ 0.9 at 5 min and ≥ 0.8 at 10 and 15 min. The bulk of data, including the results of the present study, suggests that HPI working on non-invasive arterial pressure waveform has a similar performance of HPI working on invasive arterial pressure waveform. The small difference in sensitivity and specificity can be due to factors such as different sample size, surgery population and kind of surgery.

The non-invasive ClearSight system uses an implementation of vascular unloading technique or volume clamp method (Fig. 1, panel B). Physio-Cal algorithm is used for periodic calibration of the unloaded volume of the finger artery: this ensures reliable continuous estimation of arterial pressure even in disparate clinical situations [23–27]. The ClearSight has good precision in tracking subtle changes in pressure waveforms, suggesting that non-invasive waveform features are very close to those of invasive waveforms [24–27].

In the present study ROC analysis also showed that the performance of ΔMAP in predicting hypotension at 5, 10, and 15 min was low compared with HPI, in agreement with the findings of Hatib and Davies on invasive arterial waveforms [16, 17].

Our results enable prediction, and potentially also prevention of hypotension in a much larger patient population exposed to the risk of IOH but in which arterial cannulation is seldom used. Moreover, arterial cannulation imposes the risk of several complications (nerve damage, infection, pseudoaneurysm), and is therefore currently restricted to selected surgical cases [28]. Given that the majority of gynaecologic surgical procedures are carried out with non-invasive monitoring, these findings may broaden the advantage of reducing the extent of IOH to many patients.

In our opinion there are also translational aspects in this study to be considered, that rely above all on the fact that engineering advances and machine learning models applied to medical research as in the case of HPI, could facilitate the physicians to treat IOH always more as a potentially modifiable risk factor for major postoperative complications whose association although well established, still needs further characterization in terms of clinical outcomes [22, 29]. Furthermore, easiness of interpretation of the HPI represents another important feature since it could perhaps allow non-invasive continuous hemodynamic monitoring to extend its use outside operating rooms and intensive care units also to medical and nursing staff non-skilled in invasive monitoring.

This study has several limitations. The first is the small sample size, with heterogeneous characteristics of kind of GOS. The minimum amount recommended for external validation of a multivariable model such as HPI is 100 (in this case hypotensive) events, and we analysed 135 events [30]. The wide spectrum of surgical interventions demonstrated that HPI working on ClearSight was effective in predicting IOH, however a larger study, with sub-cohorts of specific gynaecologic interventions would be required to investigate the role of HPI in several clinical situations. Moreover, our results don’t show a degrading of the AUC, specificity, sensitivity and NPV over time, in fact the values at 15 min are higher than at 10 min: probably this unusual finding could be due to the relatively small sample size.

The second limitation is due to lacking information on detailed doses of vasopressors and fluids that were administered to treat hypotensive events, so we are unable to determine the correlation between the HPI value and the interventions performed. Unfortunately, this is inherent in the retrospective nature of this data analysis: vasopressors and fluids were administered in response to hypotensive events, and we are unable to determine the correlation between the HPI value and the interventions performed to treat hypotension. This may have limited the duration of some hypotensive events, because only those lasting more than 1 min have been considered. As stated by Davies et al., this would lead to an increased false positive rate for the prediction index and could reduce the predictive ability of HPI [17]. Only prospective randomized clinical trials will be able to demonstrate whether such an HPI-based hemodynamic management would reduce the incidence and/or duration of IOH in GOS. Finally, as previously stated, HPI has been demonstrated to predict hypotension, but not clinical outcomes. Analyzing complications of IOH and associated adverse clinical endpoints will help to assess the clinical effects of incorporating HPI into anaesthesiologic practice.

## Conclusion

In conclusion, the HPI working with non-invasive ClearSight finger cuff provides real time and accurate prediction of impending arterial hypotension in patients undergoing GOS.

## Supplementary Information

Below is the link to the electronic supplementary material.

Supplementary file1 (DOCX 18 kb)

## Data Availability

The datasets used and/or analysed during the current study are available from the corresponding author on reasonable request.
