# Supplementary material for: Hypotension Prediction Index with non-invasive continuous arterial pressure waveforms (ClearSight): clinical performance in Gynaecologic Oncologic Surgery
Source: J Clin Monit Comput. 2021 Oct 7;36(5):1325–32. doi: 10.1007/s10877-021-00763-4 (PMC8496438; doi:10.1007/s10877-021-00763-4)
Supplement: Supplementary file 1 — Supplementary file1 (DOCX 18 kb) [file 10877_2021_763_MOESM1_ESM.docx]

Supplemental table 1

|  | AUC [95% CI] | Sensitivity [95% CI] | Specificity [95% CI] | PPV [95% CI] | NPV [95% CI] | Threshold |
| --- | --- | --- | --- | --- | --- | --- |
|  |  |  |  |  |  |  |
| **5 min** | | | | | | |
| Present study | 0.93 [0.89,0.97] | 0.86 [0.78,0.93] | 0.86 [0.77,0.94] | 0.88 [0.77,0.96] | 0.82 [0.70,0.91] | 44,1 |
| Maheshwari (combined)^18^ | 0.93 [0.91,0.95] | 0.86 [0.82,0.89] | 0.86 [0.82,0.89] |  |  | 30 |
| Wijnberge^19^ | 0.93 [0.92,0.94] | 0.866 [0.841,0.892] | 0.855 [0.833,0.876] | 0.882 [0.857,0.907] | 0.836 [0.805,0.867] | 34 |
| Hatib, internal cohort^16^ | 0.97 [0.971,0.977] | 0.918 [0.897,0.94] | 0.922 [0.903,0.94] | 0.888 [0.879,0.897] | 0.944 [0.938,0.949] | 41 |
| Hatib, external cohort^16^ | 0.95 [0.933,0.961] | 0.868 [0.836,0.899] | 0.885 [0.849,0.92] | 0.932 [0.91,0.953] | 0.786 [0.743,0.829] | 39 |
| Davies^17^ | 0.926 [0.925,0.926] | 0.858 [0.858,0.859] | 0.858 [0.858,0.859] | 0.889 [0.888,0.89] | 0.817 [0.816,0.819] | 40 |
| Shin^21^ | 0.9 [0.853,0.949] | 0.84 [0.777,0.905] | 0.84 [0.709,0.968] |  |  |  |
|  |  |  |  |  |  |  |
| **10 min** | | | | | | |
| Present study | 0.9 [0.83,0.97] | 0.82 [0.71,0.92] | 0.83 [0.71,0.93] | 0.79 [0.56,0.93] | 0.85 [0.73,0.94] | 41,6 |
| Maheshwari (combined)^18^ | 0.9 [0.87,0.93] | 0.83 [0.79,0.86] | 0.83 [0.79,0.86] |  |  | 28 |
| Wijnberge^19^ | 0.91 [0.9,0.92] | 0.837 [0.801,0.874] | 0.834 [0.784,0.884] | 0.789 [0.75,0.828] | 0.874 [0.845,0.902] | 32 |
| Hatib, internal cohort^16^ | 0.95 [0.95,0.96] | 0.893 [0.872,0.914] | 0.895 [0.873,0.917] | 0.767 [0.753,0.781] | 0.956 [0.951,0.961] | 38 |
| Hatib, external cohort^16^ | 0.92 [0.896,0.943] | 0.842 [0.796,0.888] | 0.843 [0.802,0.884] | 0.836 [0.794,0.878] | 0.848 [0.808,0.888] | 37 |
| Davies^17^ | 0.895 [0.894,0.895] | 0.817 [0.816,0.818] | 0.817 [0.816,0.818] | 0.792 [0.791,0.794] | 0.836 [0.835,0.838] | 37 |
| Shin^21^ | 0.83 [0.75,0.905] | 0.79 [0.698,0.881] | 0.74 [0.588,0.896] |  |  |  |
|  |  |  |  |  |  |  |
| **15 min** | | | | | | |
| Present study | 0.95 [0.89,0.99] | 0.85 [0.73,0.94] | 0.85 [0.74,0.95] | 0.75 [0.43,0.92] | 0.91 [0.81,0.98] | 44,3 |
| Maheshwari (combined)^18^ | 0.84 [0.79,0.88] | 0.75 [0.71,0.8] | 0.75 [0.71,0.8] |  |  | 22 |
| Wijnberge^19^ | 0.9 [0.89,0.91] | 0.817 [0.771,0.863] | 0.823 [0.735,0.911] | 0.703 [0.653,0.754] | 0.897 [0.87,0.924] | 31 |
| Hatib, internal cohort^16^ | 0.95 [0.94,0.952] | 0.875 [0.851,0.899] | 0.873 [0.848,0.898] | 0.672 [0.656,0.689] | 0.959 [0.954,0.964] | 36 |
| Hatib, external cohort^16^ | 0.91 [0.885,0.944] | 0.836 [0.782,0.89] | 0.833 [0.789,0.878] | 0.74 [0.679,0.801] | 0.9 [0.865,0.934] | 36 |
| Davies^17^ | 0.879 [0.879,0.880] | 0.806 [0.805,0.807] | 0.806 [0.805,0.807] | 0.726 [0.724,0.729] | 0.865 [0.864,0.866] | 36 |
| Shin^21^ | 0.83 [0.746,0.911] | 0.79 [0.684,0.89] | 0.74 [0.588,0.896] |  |  |  |

Supplemental Table 1. Receiver operating characteristic analysis for HPI (Hypotension Prediction Index) to predict hypotension 5, 10 and 15 minutes before its occurrence across different studies. Of note, the first 3 studies of each section (present study, 18, 19) applied HPI with non-invasive continuous arterial pressure waveform monitoring, while in the remaining studies (16, 17, 21) HPI was applied with invasive arterial pressure waveform. AUC: area under the curve. CI: confidence interval. PPV: Positive Predictive Value. NPV: Negative Predictive Value.
